# A universal color curve for roasted arabica coffee

**DOI:** 10.1038/s41598-025-06601-w

**Published:** 2025-07-07

**Authors:** Laudia Anokye-Bempah, Timothy Styczynski, William D. Ristenpart, Irwin R. Donis-González

**Affiliations:** 1 Department of Biological and Agricultural Engineering, University of California Davis, 3024 Bainer Hall, Davis, CA 95616 USA; 2 Coffee Center, University of California Davis, Davis, CA 95616 USA; 3 Bridge Coffee Co., Marysville, CA 95901 USA; 4 Department of Chemical Engineering, University of California Davis, Davis, CA 95616 USA

**Keywords:** Coffee roasting, Roast profiles, Roast color, CIELAB color space, Mixed-effects regression, PRISMA, Chemical engineering, Process chemistry

## Abstract

Color is a key indicator of quality and roast level in coffee beans. Surprisingly, little is known about the effect of different “roast profiles,” i.e., the temperature versus time inside the roaster, on the dynamics of color during roasting, with most prior work focused on laboratory-scale roasters with little control over the roast profile. Here, we investigate seven roast profiles of the same total duration but varying dynamics inside a 5 kg commercial drum roaster, using coffee from three origins. We show that despite the dramatic differences in roast profiles and coffee origins, the bean color always maps onto a “universal roasted coffee color curve” when plotted in the L*a*b* color space. This universal color curve was modeled using polynomial mixed-effects regression and validated through a systematic review of existing literature following the PRISMA protocol to demonstrate its broad applicability. Although the dynamics of roast color development varied with roast profiles, the coffees always had approximately the same L*a*b* values at significant roast milestones, including color change, first crack, and second crack. We discuss how these results provide insight into color measurements and how they can quantitatively inform roast-level standards in the coffee industry for both real-time and post-roast applications.

**Supplementary Information:**

The online version contains supplementary material available at 10.1038/s41598-025-06601-w.

## Introduction

Color is one of the most important parameters used in coffee characterization. In the coffee industry, coffee bean color serves as a valuable indicator of roast level and plays a crucial role in quality assessment and consumer preferences^1–3^. Moreover, changes in bean color during roasting have been correlated with variations in other physicochemical properties, including acrylamide content^4^, aroma composition^5^, antioxidant activity, and volatile compounds^6,7^, as well as chlorogenic acid and caffeine content^8^.

Generally, roast color is determined by visual (human) inspection or by comparison with reference tiles such as the Specialty Coffee Association of America (SCAA) color disks^9^. Although widely used, visual assessments are subjective and can be influenced by numerous factors such as illumination, sample size, surrounding color, and the angle of observation^10^. Consequently, color-measuring instruments, like spectrophotometers and colorimeters, have been developed to provide standardized conditions for accurate and consistent measurements. Spectrophotometers measure the spectral reflectance (or transmittance) of whole or ground coffee samples at different visible spectrum wavelengths (380 nm to 780 nm). Readings are presented as reflectance spectra or converted into standard roast level measurement scales such as the Agtron classification system^10,11^. Colorimeters, on the other hand, quantify color based on the three-component theory of color vision, using three sensors to mimic human eye perception of color. Thus, colorimeters measure the intensity of light reflected from or transmitted through a sample and convert these measurements into X-Y-Z tristimulus values, which are then translated into standard color spaces such as RGB, CIE L*a*b*, CIE L*u*v*, CIE Yxy, or CIE LCH^12^. The CIELAB or L^∗^a^∗^b^∗^ color space, which is a color standard implemented by the Commission Internationale de l’Eclairage’ (CIE, 1976), is widely used because it provides a perceptually uniform color space, where the Euclidean distance between two different colors corresponds approximately to the color difference perceived by the human eye^10^. This system describes color by three coordinates: L*, a*, and b*. The L* coordinate represents the luminance or lightness component, ranging from 0 to 100 (black to white), while coordinates a* (from green to red) and b* (from blue to yellow) are two chromatic components often cited to range from − 120 to 120 in practical applications. However, these ranges are not absolute and can extend beyond these values, depending on the implementation and measurement equipment^10,13^.

It is well known that during roasting, the color of coffee beans progressively changes to yellow, brown, dark brown, and finally to black^14^. Various studies have investigated the impact of roasting on coffee color^15–24^. However, much less data exists regarding how the color changes as a function of specific “roast profiles,” the term used in the coffee industry to denote the temperature versus time measured inside the roaster. Most work has instead focused on coffee roasted in ovens held at constant temperatures. Early work by Little and Mackinney (1956) examined the impact of five roasting temperatures (150–200 °C) and various coffee origins on the lightness (L*) of roasted coffee. They reported a consistent decrease in L* values with increasing roasting temperature and similar rates of change across different coffee origins^25^. Schenker (2000) investigated the effects of isothermal roast profiles, including high-temperature short-time (HTST, 260 °C,180 s) and low-temperature long-time (LTLT, 220 °C, 720 s) on L*a*b* color coordinates during roasting. They found that higher temperatures led to faster changes in roast color, with consistent color pathways observed across the different roast profiles^26^. Wang and Lim (2012) further examined L* values at key roast stages (green coffee, first and second crack, 48 s after first and second crack) using four isothermal roast profiles (210–240 °C) and reported significant changes in L* values up to second crack^27^. Similarly, Pramudita et al. (2017) and Mehaya and Mohammad (2020), using drying ovens, investigated the effects of isothermal temperatures (ranging from 140 °C to 300 °C) and time (10 min to 24 h) on coffee color formation. Both studies found that higher temperatures resulted in a faster decrease in L* values, with higher temperatures consistently producing lower final L* values^18,28^. Other studies using isothermal roasting have corroborated these results^1,16,24^.

Notably, these studies only measured color at the beginning and end of roasting or at infrequent intervals, providing an incomplete picture of the color changes throughout the roasting process. More importantly, the above studies used isothermal roasting or oven-drying methods, which do not reflect common practice in commercial-scale roast profiles^26^. Specifically, it remains unclear how industry-standard roast profiles, particularly those involving large batch sizes (> 1 kg) and significant temperature fluctuations over time, influence the dynamics of coffee color during roasting. These studies also focused on a limited set of isothermal profiles, such as HTST and LTLT, which do not reflect the wide range of profiles used in the coffee industry. Furthermore, the relationships among the L*a*b* color coordinates during commercial-scale roasting have yet to be systematically investigated. The most detailed work correlating L*a*b* color coordinates focuses on isothermal conditions in a drying oven for up to 24 h with a small sample size of 5 g^18^. It remains unclear how industry-standard roast profiles might influence these correlations or how they affect different types of green coffee from various origins or processed with different methods.

The goal of this study was to evaluate how different roast profiles and coffee origins impact the changes in coffee color during roasting in a representative commercial-scale roaster. Additionally, we sought to evaluate the relationship between the L*a*b* color coordinates during roasting. Toward this goal, we first examined the impact of seven very different roast profiles on the color of a single-origin coffee. The total duration of each roast was held constant, but we varied the energy inputs to yield different roast profiles. Samples were collected every minute from the roaster to measure the L*a*b* values. Next, we assessed how the color changes depended on the origin used to process the coffee beans, examining a smaller subset of roast profiles. Lastly, we investigated the correlations between the L*a*b* color coordinates and conducted a systematic review and meta-analysis to compare our results to existing literature. An important finding is that regardless of the roast profile or coffee origin, the color of coffee during roasting always follows a “universal roasted arabica coffee color curve” when plotted in the L*a*b* color space.

## Materials and methods

### Overview of the experimental design

The data presented here were gathered during a series of experiments conducted at the UC Davis Coffee Center from July to December 2022 to systematically investigate the impact of roast profiles on different important coffee metrics. Details regarding the specific roast profiles and their corresponding impact on titratable acidity have already been published in Anokye-Bempah et al.^29^. Here, we focus specifically on color changes in arabica coffee during roasting.

In brief, we examined seven roast profiles— “fast start” (FS), “slow start” (SS), “medium” (MD), “production” (PR), “exaggerated flick” (EF), “negative rate of rise” (NR), and “extended Maillard” (EM)—using a 5-kg commercial batch roaster (P5 model 2, Probat GmbH, Emmerich am Rhein, Germany). Each roast lasted 16 min, and samples were collected at one-minute intervals, yielding 17 total samples (16 samples during the roast plus its corresponding green coffee sample). All seven roast profiles were performed on a washed Ugandan coffee. We further investigated a smaller subset of roast profiles (FS, SS, and EM) with two additional coffees: a washed Indonesian coffee and a honey-processed Central American coffee. Each roast was performed in triplicate to allow for complete statistical analysis. Thus, we performed 39 experimental roasts (7 × 3 for the roast profiles experiment and 2 × 3 × 3 for the coffee origins experiment), with 17 samples per roast, yielding 663 samples. Subsequently, all samples were ground and assessed using colorimetric measurements in the L*a*b* color space.

### Green coffee and roast profiles

Green coffee (*Coffea arabica*) beans from three different origins (geographical locations) were used: a washed Ugandan coffee from Sipi Falls (USF), a washed Indonesian coffee from Sumatra (SUM), and a honey-processed Central American coffee from Ataco, El Salvador (ELS), chosen for their very different taste profiles. Before the roasting experiments, the green coffees were packed and labeled into smaller 1 kg jute sacks (Model No. S-8423, Uline, Pleasant Prairie, WI, USA), fastened with a zip tie, and stored in an environmental chamber (Caron Inc., model 7000–25, Marietta, OH, USA), with conditions set to mimic typical industry warehouse storage conditions of 25 °C and 60% relative humidity. After a minimum 10-day storage period, the coffee beans reached a 10.5 ± 0.5% wet basis moisture content, as determined by the method outlined in Anokye-Bempah et al.^30^.

**Fig. 1:**
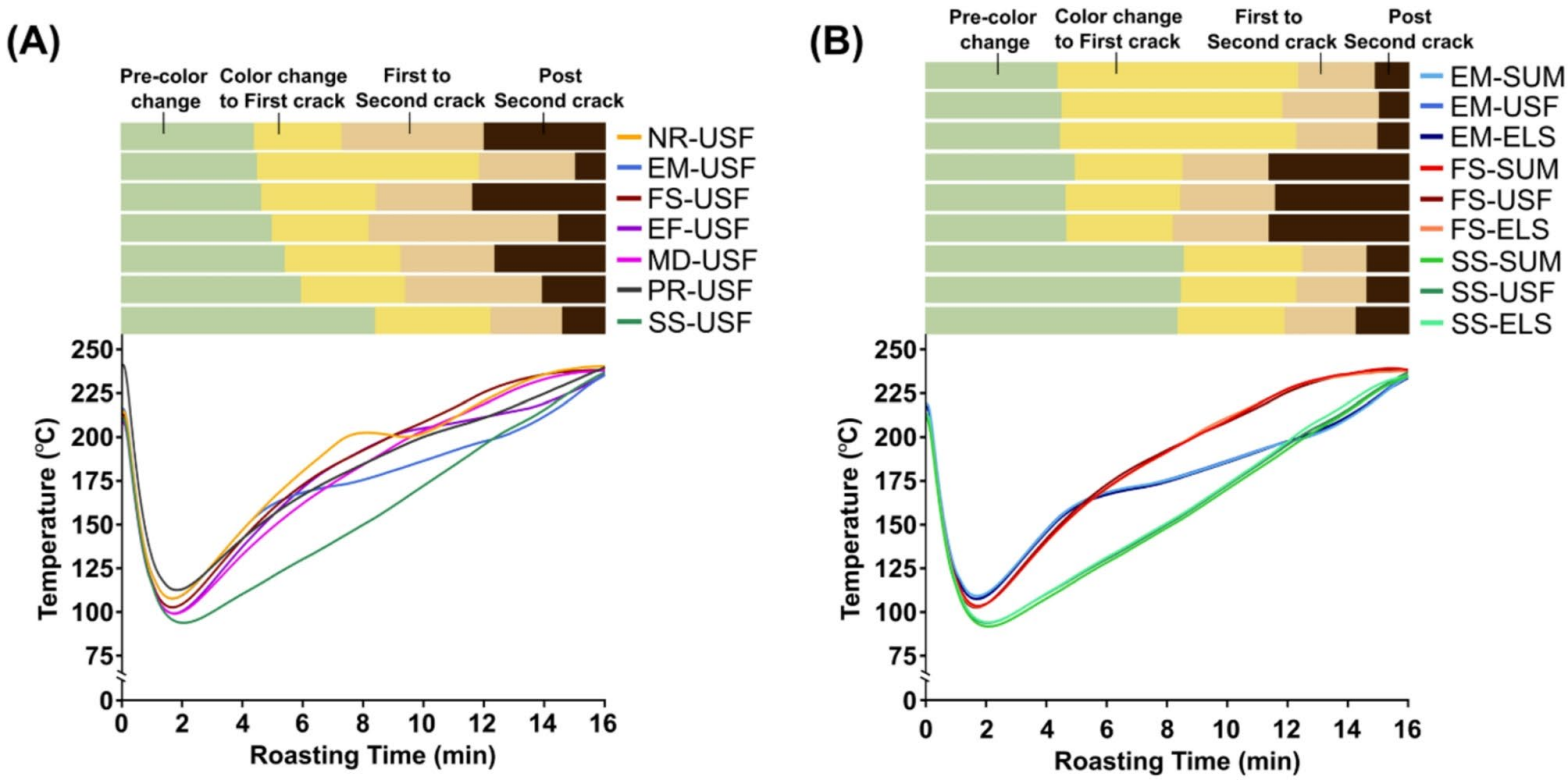
(**A**) Roast profiles (fast start (FS), slow start (SS), medium (MD), production (PR), exaggerated flick (EF), negative rate of rise (NR), and extended Maillard (EM)) used to roast the washed Ugandan coffee (USF). (**B**) Roast profiles used to roast the three different green coffees; USF, honey-processed Central American coffee (ELS), and washed Indonesian coffee (SUM). Colored lines represent temperature vs. time in the roast drum, with each line depicting the mean of three replicates per roast profile. The colored rectangles above each subfigure denote the roast milestones.

Key changes in each profile are summarized in Table 1, achieved by adjusting energy dynamics and heat intensity through gas flow and airflow. Before each roast, the roaster was preheated to 210 ± 5 °C for 30 min to stabilize the drum temperature. All roasts had a similar starting and final temperature of 215 ± 8 °C and 237 ± 2 °C, respectively, and lasted a total of 16 min to allow sufficient time to investigate subtle changes in color from the green coffee stage to the burnt/charred coffee stage. Each roast had three milestones: color change (which is the stage at which the roaster operator observes that the bean color has appreciably altered from its original color), first crack, and second crack, which were qualitatively denoted by an experienced roaster based on visual and auditory cues. The first set of experiments examined all seven roast profiles using the washed Ugandan (USF) coffee. Subsequent analysis of the collected roast profile data revealed that the FS, SS, and EM profiles were the most distinct, so these profiles were selected to roast the washed Indonesian (SUM) and honey-processed Central American (ELS) coffees as detailed in Anokye-Bempah et al. (2024)^29^. Figure 1a summarizes the seven roast profiles for the USF, while Fig. 1b shows the profiles for SUM and ELS coffees.

**Table 1:** Roast profile parameters collected during roasting, including the initial and final rate of rise (RoR), as well as the duration and mean RoR for the major roast milestones. The initial RoR reflects the highest positive RoR immediately after the turning point, or when the coffee beans and the roaster temperature equilibrate on each roast curve. The final RoR indicates the RoR during the last minute of the roast. Adapted from Anokye-Bempah et al.^29^.

Coffee type	Roast profile	Initial RoR (°C/30s)	Final RoR (°C/30s)	Roast milestones							
				Pre-color change		Color change to first crack		First to second crack		Post second crack	
				Duration (min)	Mean RoR (°C/30s)	Duration (min)	Mean RoR (°C/30s)	Duration (min)	Mean RoR (°C/30s)	Duration (min)	Mean RoR (°C/30s)
Ugandan washed (USF)	Fast start (FS): High initial heat, followed by decelerating roast energy	10.15	0.08	5.0	7.97	3.5	5.39	3.5	4.39	4.0	1.58
	Slow start (SS): Low initial heat, followed by accelerating roast energy	5.26	4.56	8.5	5.07	4.0	5.79	3.5	5.2	1.0	4.83
	Medium (MD): Characteristics fall between FS and SS profiles	9.11	0.63	5.5	7.92	4.0	5.51	3.0	3.89	3.5	2.14
	Production (PR): Profile achieved by maintaining constant roast energy	8.19	3.69	5.0	7.09	4.5	4.56	4.5	3.13	2.0	3.75
	Exaggerated flick (EF): Sudden RoR increase after first crack	10.37	4.93	5.0	7.86	3.5	5.97	6.0	2.13	1.5	4.32
	Negative rate of rise (NR): Mimics gas flow loss, especially around first crack	10.11	0.32	4.5	7.98	2.5	7.72	5.0	2.58	4.0	2.46
	Extended maillard (EM): Replicates a ‘baked’ profile with rapid pre-color change and extended color change to first crack phase	10.20	5.32	4.5	7.88	7.5	2.80	2.0	3.60	2.0	5.83
Central American honey processed (ELS)	Fast start (FS)	9.91	0.37	5.0	7.56	3.5	5.68	3.0	4.40	4.5	1.62
	Slow Start (SS)	5.33	1.89	8.5	4.76	3.5	5.90	2.5	5.52	1.5	3.29
	Extended maillard (EM)	10.01	4.87	4.5	7.67	7.5	2.90	3.0	4.23	1.0	5.32
Indonesian washed (SUM)	Fast Start (FS)	9.96	0.01	5.0	8.76	4.0	5.45	2.5	4.46	4.5	1.68
	Slow start (SS)	5.07	3.72	8.5	4.72	4.0	5.72	2.5	5.49	1.0	4.23
	Extended maillard (EM)	9.90	5.43	4.5	7.51	7.5	2.79	3.0	4.18	1.0	6.02

### Sampling procedure

During each 16-minute roast, we collected 17 coffee bean samples, each weighing approximately 13 g, using the roaster sample trier. The collected samples were immediately weighed and divided into two separate 50-ml tubes (Falcon, Corning Inc., NY, USA), Tube A and Tube B. Each tube A sample contained approximately 8 g, and each tube B sample contained approximately 5 g; the tube B samples were reserved for moisture and water activity measurements not reported here. Immediately after placing the approximately 8 g sample into tube A, the entire tube was rapidly cooled in liquid nitrogen (N_2_) for about 15 s. The tube was then temporarily stored in a cooler with dry ice to transport to the Postharvest Engineering Laboratory at the Biological and Agricultural Engineering Department at UC Davis for grinding in a water-cooled laboratory mill (KN 295 Knifetec™, FOSS Analytics, Hillerød, Denmark) and subsequent colorimetric measurements. Particle size analysis was performed on two representative ground samples from the MD roast profile: sample ten, which was collected during first crack, and sample thirteen, which was collected during second crack, using a Beckman Coulter Particle Size Analyzer (LS 13 320 series; Beckman Coulter, Inc., Spain) according to the method described in Panuska et al.^31^. Sample ten had a particle size range of 25–1363 μm, with a median particle size (D50) of 393 μm, while sample thirteen had a range of 25–553 μm, with a median particle size (D50) of 96 μm.

### Color measurements

The color of each ground sample was measured using a HunterLab ColorFlex EZ Spectrophotometer (ColorFlex EZ, Hunter Associates Laboratory Inc, Reston, VA, USA) at a 2° observation angle and under D65 standard illumination using non-polarized diffuse light. Results were expressed in the CIELAB color space. Each sample (~ 8 g) was placed in an Opti-glass cylinder, and the L*a*b* values were measured. The instrument was standardized against a white and black calibration tile after every 17 measurements. Measurements were performed in triplicate for each sample replicate, resulting in 9 L*a*b* measurements per sample. To compare our color measurements with existing studies, the mean color difference (ΔE*) was calculated as the minimum Euclidean distance between two points in the CIELab color space Eq. (1):

1 $$\:\triangle\:E{\text{*}}=\:\sqrt{{({L{\text{*}}}_{2}-{L{\text{*}}}_{1})}^{2}+{({a{\text{*}}}_{2}-{a{\text{*}}}_{1})}^{2}\:+{({b{\text{*}}}_{2}-{b{\text{*}}}_{1})}^{2}}  $$

Where L*_1_, a*_1_, and b*_1_ represent the L*a*b* values from other publications, and L*_2_, a*_2_, and b*_2_ represent the corresponding closest points on our regression curve (described in Sect. 3.2). According to Hunt (1991), ΔE* values indicate perceptual color changes and range from 0 to 100. A ΔE* value of 0 to 2 signifies no perceptible difference to the human eye, values between 2 and 10 indicate differences that are noticeable at a glance, and values above 10 suggest distinct but similar colors^10^. Representative calculations using the more complicated ΔE*_00_ formulas^10^ yielded a negligible difference from Eq. (1).

Representative samples from the seven roast profiles of the USF coffee were photographed with a computer vision system to qualitatively capture color images that reflect changes in the coffee during roasting. The system comprised a color camera (Basler a2A3840-45ucPRO 8.3MP, Basler AG, Ahrensburg, Germany) with a 5MP lens (Basler C125-0418-5 M, Basler AG, Ahrensburg, Germany), mounted within the circular viewing aperture of a wide linear diffuse light (Model DL067A-18, Advanced Illumination, Rochester, VT, USA), as shown in Supplementary Figure S1. This system was designed and assembled at the Postharvest Engineering Laboratory in the Biological and Agricultural Engineering Department at UC Davis to provide uniform illumination (irradiance: 28 W/m^2^, illuminance: 10 Klux) to capture high-quality color images under controlled conditions. Images were captured using a lens aperture of f/1.8, an exposure time of 4.86 ms, a gain of 69.5 dB, and a white balance set to off. The images, acquired at maximum resolution (Bpp24, 8.3MP) with Pylon Viewer software (V8; Basler AG, Ahrensburg, Germany) were stored uncompressed in JPEG format until further analysis.

**Fig. 2:**
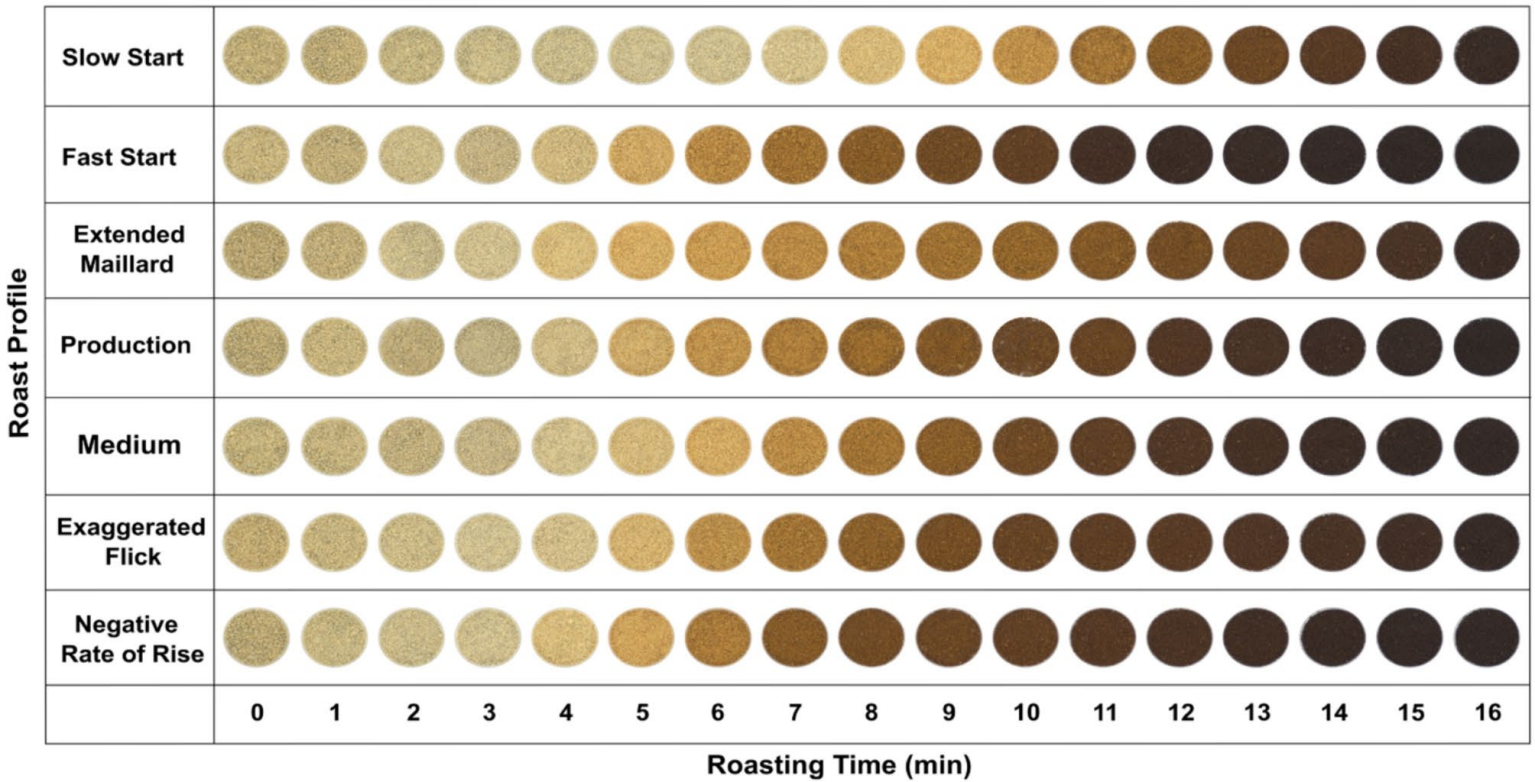
Pictures showing the color of the ground coffee samples for the seven roast profiles using the washed Ugandan coffee (USF).

### Color data extraction (meta-analysis) from existing literature

To compare our color measurements with existing studies, we conducted a systematic review following the Preferred Reporting Items for Systematic Reviews and Meta-Analyses (PRISMA) guidelines for literature search and data extraction^32^. Relevant studies were sourced from Google Scholar, Web of Science, and the University of California Library catalog using the keywords ‘coffee roasting color,’ ‘coffee roasting L*a*b*,’ ‘coffee color curve,’ and ‘coffee roast profiles.’ Searches were limited to English-language peer-reviewed journal articles, conference papers, and theses on color dynamics during coffee roasting published before December 2024. Studies were included in the review if they (1) examined the effects of roast profiles or roast degrees on coffee color using Arabica or Robusta (*C*. *canephora*) coffee beans from any origin or postharvest processing method; (2) provided ground coffee color data using the L*a*b* color space; and (3) provided detailed descriptions of their roasting procedures and roast profiles. Studies that only reported whole bean color measurements were excluded, as whole bean color significantly differs from ground coffee color^1,33^. For context, we included one study on color changes during bread baking to compare our findings with another Maillard reaction process^34^. Additionally, seven studies were included for completeness despite lacking detailed roasting method descriptions or color measurement procedures, as they still reported potentially relevant color data. Supplementary Figure S2 summarizes the study identification process in the form of a PRISMA flow diagram. In total, data from 20 different publications were collected and compared with our experimental data (Table 4).

### Statistical analysis and data visualization

All statistical analyses and data visualization were performed using R version 4.4.1^35^. The L*a*b* values were averaged over three measurement replicates within each sample replicate. Two-way mixed ANOVAs were used to determine the statistical significance of differences among roast profiles and between coffee origins for the L*a*b* color coordinates. Additionally, the median of the major roast milestones (color change, first crack, and second crack) was calculated, and a one-way ANOVA was used to determine significant differences in the L*a*b* values at these milestones for all roast profiles and coffee origins. Whenever the ANOVA test was significant, differences were inferred by applying a post hoc Tukey Honest Significant Difference (HSD) test. A type I error rate (α) of 0.05 was used as the threshold for reporting significant differences. Subsequently, polynomial mixed-effects regression models were used to analyze the relationships between the L*a*b* color coordinates. The overall quadratic relationships between a* vs. L* and b* vs. L* were modeled as fixed effects, while random intercepts and slopes were included for each roast profile to account for the repeated measures. The models were estimated using the `lme` function from the `nlme` package^36^ in R with restricted maximum likelihood (REML) estimation. Model assumptions were verified through residual analyses.

## Results

### Effect of roast profiles and green coffee origin on color

Figure 2 shows representative photos of the ground coffee samples across the seven roast profiles. For all tested roast profiles, the bean color progressively changed from its initial ‘green coffee’ color to yellow, brown, dark brown, and finally to black (Fig. 2). Although often referred to as ‘green coffee,’ the color of coffee beans before roasting can be categorized as bluish, greenish, grayish-green, olive-green, whitish, yellowish, or brownish, as defined by ISO (2005)^37^. In our case, the color was closer to a grayish yellow. The closest named centroid in the National Bureau of Standards (NBS) color dictionary is grayish-greenish-yellow^38^. While the overall trend of color changes was consistent across roast profiles, the different roast profiles strongly affected the color dynamics. Roast profiles with a high initial rate of rise (RoR)—the rate of temperature increase per 30 s—such as the FS profile, demonstrated faster color changes compared to roast profiles with a low initial RoR, like the SS profile (Fig. 2). Similarly, roast profiles with a medium initial RoR, such as the MD and PR profiles, exhibited rates of color changes that fell between those of the FS and SS profiles. The general trend of higher roasting temperatures leading to faster changes in roast color qualitatively accords with previous studies^15,26,27^. Surprisingly, the NR and EF roast profiles, which are believed to be associated with common roasting defects^39 ^such as flat or baked flavors in the final product quality, yielded color profiles almost indistinguishable from the FS roast profile.

**Fig. 3:**
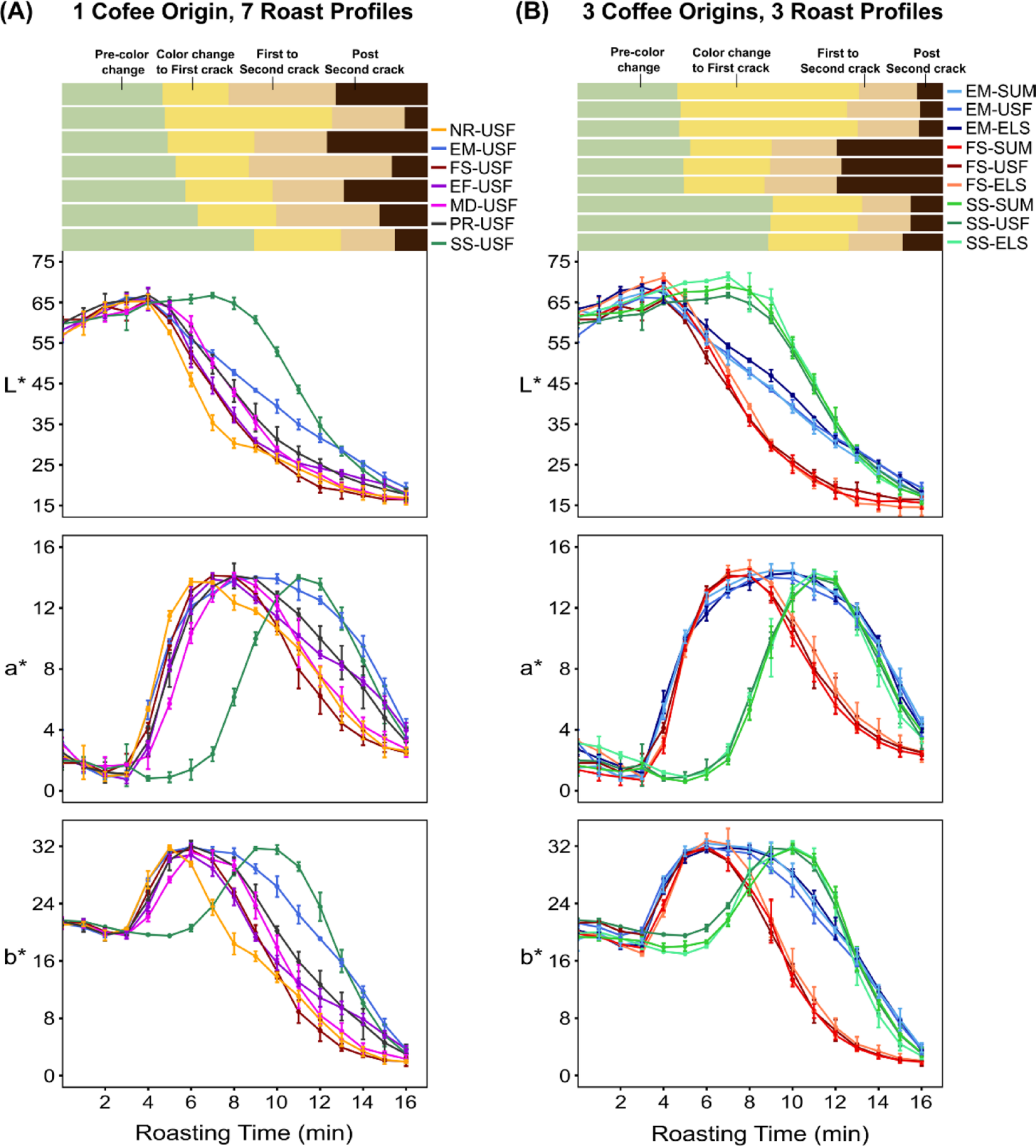
(**A**) Direct comparison of the L*a*b* values during roasting of the washed Ugandan coffee (USF) using seven different roast profiles: fast start (FS), slow start (SS), medium (MD), production (PR), exaggerated flick (EF), negative rate of rise (NR), and extended Maillard (EM). Each colored line represents a different roast profile, and the error bars indicate one standard deviation from the mean within three roasting replicates. (**B**) Direct comparison of the L*a*b* values for the three green coffees (USF, SUM, and ELS) roasted using FS, SS, and EM roast profiles. The lines with slightly different shades (e.g., dark red, bright red, and orange-red) denote the three different origins for that same roast profile, with red indicating FS, green indicating SS, and blue indicating EM profiles.

Quantitative measurements of the colors corroborated the qualitative results (Fig. 3a). The mean initial L*a*b* values, reflecting measurements across 7 × 3 = 21 sample replicates of the same green coffee, were 59.33 ± 2.3, 2.43 ± 0.7, and 21.33 ± 0.4 respectively (Fig. 3a). Consistent with the qualitative results, we observed a marginal increase in L* values from the beginning of the roast until the color change to first crack phase as the beans changed to yellow, followed by a consistent decrease in L* values till the end of the roast (as the beans turned brown and finally black). Similarly, the a* and b* values significantly increased from the beginning of the roast until the color change to first crack as the coffee beans became more red and more yellow, then decreased continuously as the beans darkened toward the end of the roast.

Two-way mixed ANOVAs performed on the L*a*b* values with time as the within-subjects factor and roast profiles as the between-subjects factor showed significant differences (α = 0.05) in the L*a*b* color coordinates based on roast profile, time, and roast profile x time interactions (Table 2). These results indicate that all three color coordinates were significantly affected by both roast profile and roasting time. Post-hoc Tukey’s HSD Test for multiple comparisons among roast profiles showed that the mean values of L*, a*, and b* (averaged over roasting time) were significantly different between FS, SS, MD, and EM (*p* < 0.001). There was no statistically significant difference between the EF and PR roast profiles (*p* > 0.05). The full Tukey HSD results, including detailed comparisons for each roast profile, are shown in (Table 3).

**Table 2:** Table of F-ratios from the mixed ANOVAs, significance indicated by * (α = 0.05), with corresponding degrees of freedom (df) and p-values (df values were corrected using Greenhouse-Geisser estimates due to a violation of sphericity indicated by Mauchly’s test).

Parameter	Factor	df	F-ratio	*P*-value
L*	Time	2.40	2708.55	< 0.001*
	Time* Roast profile	14.42	27.08	< 0.001*
	Roast profile	6.00	40.87	< 0.001*
a*	Time	3.39	1054.47	< 0.001*
	Time* Roast profile	20.37	39.50	< 0.001*
	Roast profile	6.00	63.38	< 0.001*
b*	Time	3.82	1984.36	< 0.001*
	Time* Roast profile	22.91	50.96	< 0.001*
	Roast profile	6.00	56.00	< 0.001*

**Table 3:** Post-hoc comparisons for L*a*b* color coordinates across roast profiles using Tukey’s HSD test.

		L*	a*	b*
(I) Profile	(J) Profile	Sig.	Sig.	Sig.
EF	EM	0.001	< 0.001	< 0.001
	FS	0.354	0.007	0.108
	ME	0.999	0.006	0.954
	NR	0.029	0.107	0.003
	PR	0.333	0.831	0.058
	SS	< 0.001	< 0.001	< 0.001
EM	FS	< 0.001	< 0.001	< 0.001
	ME	0.003	< 0.001	< 0.001
	NR	< 0.001	< 0.001	< 0.001
	PR	0.074	< 0.001	< 0.001
	SS	< 0.001	< 0.001	0.034
FS	ME	0.190	1.000	0.451
	NR	0.728	0.746	0.504
	PR	0.008	< 0.001	< 0.001
	SS	< 0.001	0.001	< 0.001
ME	NR	0.013	0.678	0.020
	PR	0.558	< 0.001	0.010
	SS	< 0.001	0.001	< 0.001
NR	PR	< 0.001	0.010	< 0.001
	SS	< 0.001	< 0.001	< 0.001
PR	SS	< 0.001	< 0.001	0.098

To test whether the trends shown in Fig. 3a were unique to that specific USF coffee, we repeated the measurements with two other coffees. Figure 3b shows how the L*a*b* values varied with the FS, SS, and EM roast profiles for all three green coffee origins. The overall trends for the ELS and SUM coffees are extremely similar to the USF coffee analyzed in (Fig. 3a). The L*a*b* values first increased until the color change to first crack phase, then decreased towards the end of the roast (Fig. 3b). The ANOVA results showed significant differences in b* values among the coffee origins for the SS roast profile and significant differences in L* values among the coffee origins for the EM profile (*p* < 0.05). However, there were no significant differences in the L*, a*, or b* values among the coffee origins for the FS roast profile (*p* < 0.05) (Supplementary Table S1). These results suggest that the coffee origin may affect the color dynamics during roasting, depending on the roast profile used.

**Fig. 4:**
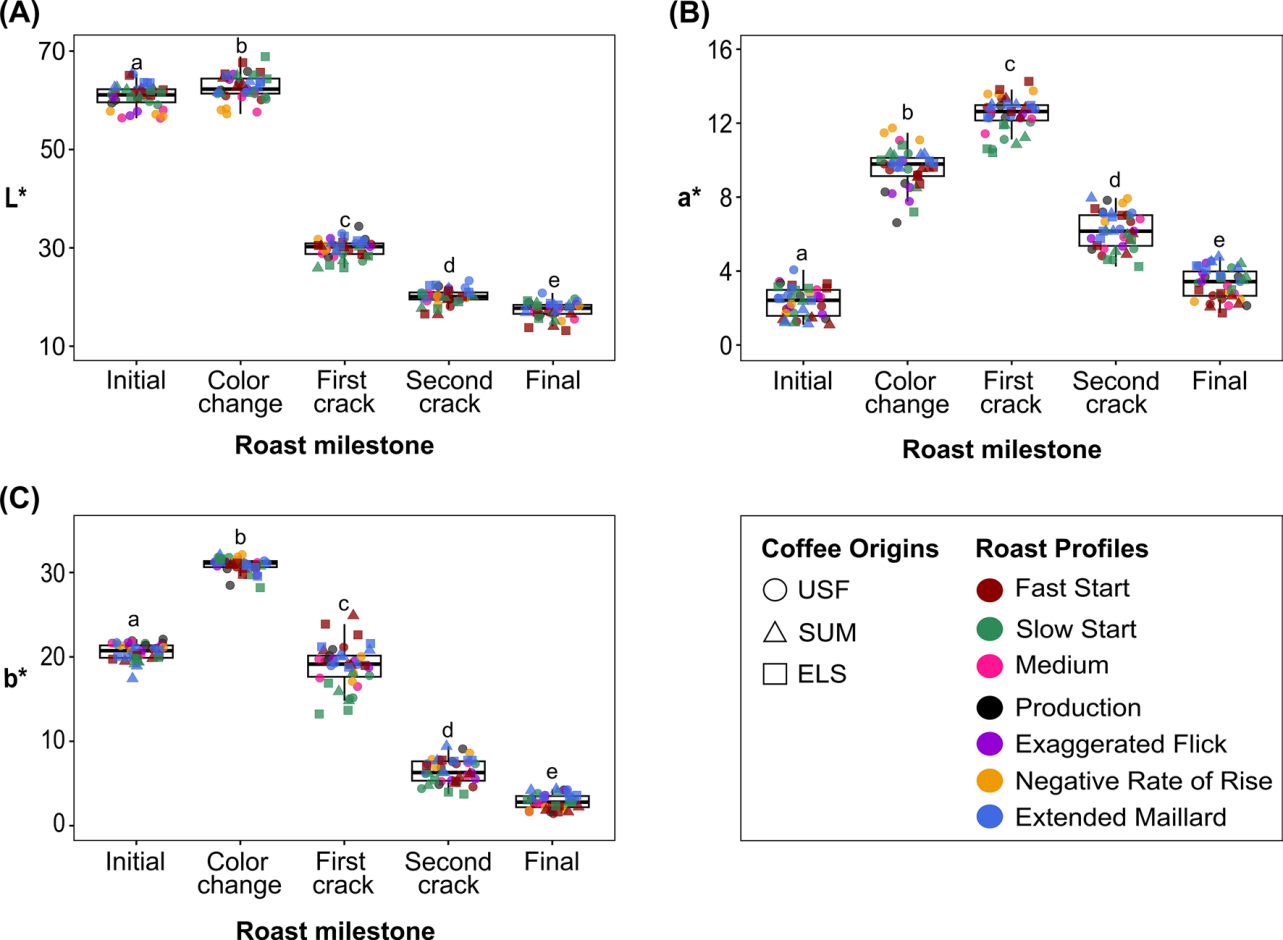
Boxplots showing the distributions of L*, a*, and b* values (**A**-**C**, respectively) at each roast milestone for all seven roast profiles and three coffee origins. The bottom and top edge of the boxes represent the 25 and 75th percentiles, respectively, the line inside the box represents the median, and the whiskers denote the range of the observed values. Lowercase letters indicate statistically significant differences among roast milestones according to Tukey HSD test.

A surprising aspect of our findings is that, regardless of the roast profile or coffee origin, we observed similar L*a*b* values at the major roast milestones: color change, first crack, and second crack. In other words, despite the differences in roast profiles and coffee origins, all samples exhibited a similar color at these major roast milestones. Figure 4 shows box plots of the L*a*b* values distribution at each roast milestone. Each data point represents the L*, a*, or b* value, with the color and shape of the data points indicating the roast profile and coffee origin, respectively. The letters above the box plots indicate significant differences in the distribution of L*a*b* values across the roast milestones, as determined by a Tukey HSD test with a p-value < 0.01. As shown in Fig. 4a, all samples had an average L* value of 62.38 ± 2.6 at color change, 29.74 ± 1.9 at first crack, 19.96 ± 1 at second crack, and 17.19 ± 1.6 at the end of the roast, irrespective of the roast profile or coffee origin. Similarly, the average a* value of all samples was 9.62 ± 1.26 at color change, 12.56 ± 0.78 at first crack, 6.41 ± 1.02 at second crack, and 3.24 ± 0.79 at the end of the roast, while the average b* value was 30.85 ± 0.86 at color change, 18.92 ± 2.53 at first crack, 6.45 ± 1.41 at second crack, and 2.84 ± 0.88 at the end of the roast (Fig. 4b,c).

### Correlations of the L*a*b* color cordinates and the coffee color curve 

**Fig. 5:**
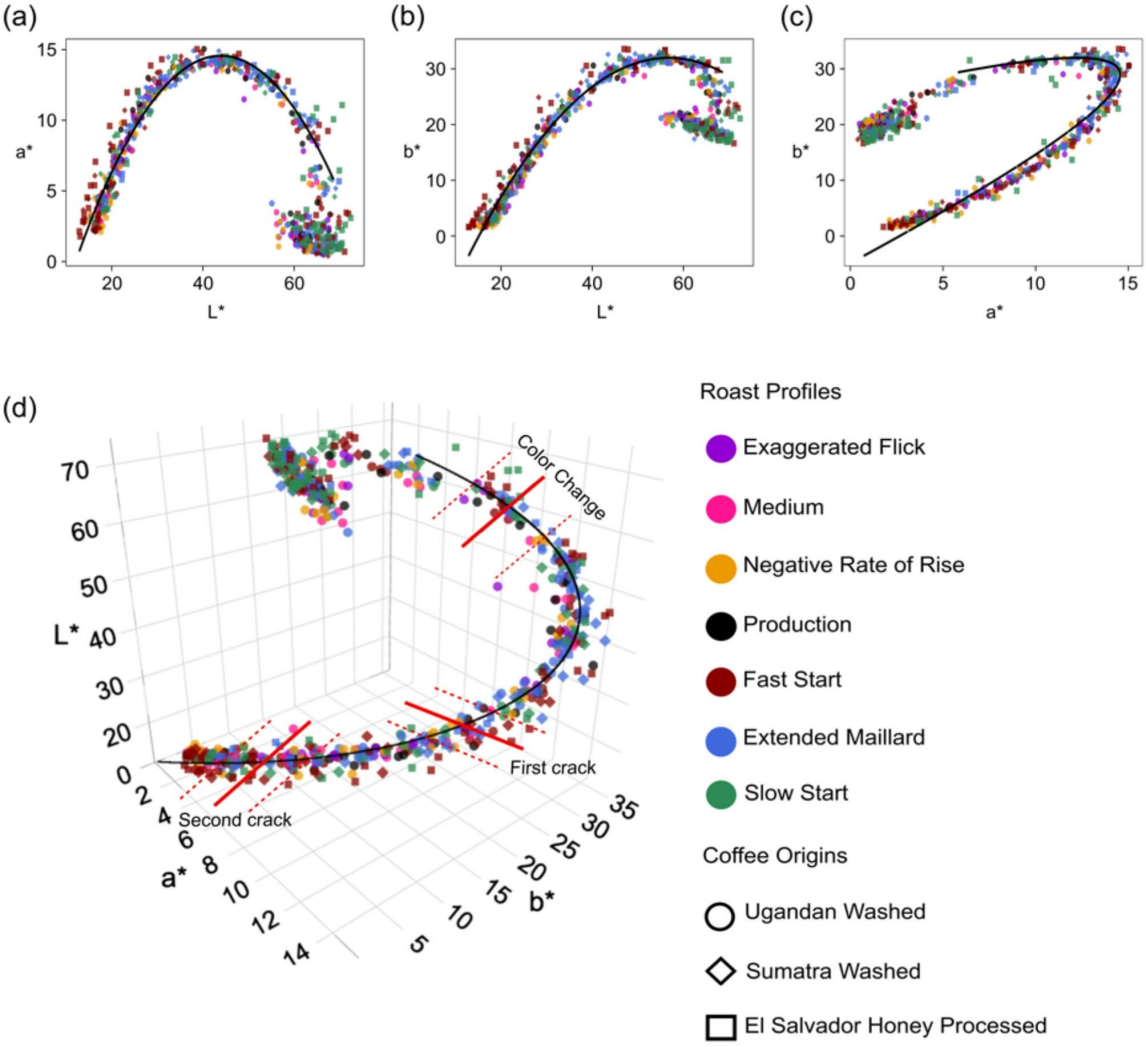
Correlations between the L*a*b* color coordinates for the seven roast profiles and three coffee origins. (**a**) a* vs. L*, (**b**) b* vs. L* (**c**) a* vs. b*, and (**d**) L* vs. a* vs. b*. Three-dimensional plot of the L*a*b* values for all roast profiles and coffee origins. The black curved lines represent second-order polynomial regression curves, with red dashed lines marking the start and end of each roast milestone, and the thick red line indicating the median of each milestone.

To determine the relationship between the L*a*b* coordinates, we performed regression analyses of a* vs. L* and b* vs. L* values across all roast profiles and coffee origins, focusing on the color change phase through to the end of each roast. Data from the pre-color change (green coffee) phase were excluded due to the high but natural variability in the color of green coffee beans. The resulting scatter plots and regression curves are shown in (Fig. 5).

A key finding in our results was that although the plots of a* vs. L* (Fig. 5a), b* vs. L*(Fig. 5b), b* vs. a*(Fig. 5c), and the 3D plot of L*a*b* (Fig. 5d) consisted of L*a*b* values obtained using different roast profiles and coffee origins, the L*a*b* values appeared to follow a single curve, starting near L* = 60 for green coffee and reaching about L* = 20 for very dark coffee. This finding suggests that regardless of the roast profile or coffee origin, the changes in coffee color during roasting follow a consistent path, which we refer to as “the universal roasted arabica coffee color curve.” To assess how closely our measured L*a*b* values followed the universal coffee color curve, we calculated the ΔE* between each data point and the nearest point on the regression curve. The average ΔE* was 1.19 ± 0.76. Specifically, 86.48% of our data points had a ΔE* < 2, 96.27% had a ΔE* < 3, and 99.53% had a ΔE* < 4. A histogram of the ΔE* values is provided in Supplementary Figure S3. The results of the polynomial mixed-effects regression analyses showed that coordinate a* explained 93.4% of the variance in L* (Marginal R² = 0.934, *p* < 0.001), with both fixed effect slopes for L* and L*^2^ being significant (β = 1.341, *p* < 0.001 for L*; β = -0.015, *p* < 0.001 for L*^2^). Similarly, coordinate b* accounted for 97.7% of the variance in L* (Marginal R² = 0.977, *p* < 0.001), with both fixed effects for L* (β = 2.244, *p* < 0.001) and L*^2^ (β = -0.020, *p* < 0.001) being significant. The fitted regression models for the a* vs. L* and b* vs. L* relationships, which describe the roasted arabica coffee color curve, are represented by the following equations:

2 $$\:{\text{a}}{\text{*}}=-14.498+1.341\left({\text{L}}{\text{*}}\right)-0.015({{\text{L}}{\text{*}})}^{2}$$

3 $$\:{\text{b}}{\text{*}}=-30.221+2.244\left({\text{L}}{\text{*}}\right)-0.020({{\text{L}}{\text{*}})}^{2}$$

Diagnostic checks, including residual analysis, confirmed the models’ adherence to the assumptions of polynomial regression, with no violations observed (See Supplementary Figure S4).

### A universal roasted coffee color curve

Next, we wanted to answer the question: How ‘universal’ is the coffee color curve suggested by our data? To address this question, our meta-analysis of the literature included colorimetric data from a wide variety of roasting conditions, some quite unusual compared to standard industry practice. Table 4 lists the 20 studies included in this analysis, an overview of each study’s experimental design, and the average minimum ΔE* between their color data and the color curve derived from our regression analysis.

Among the studies that satisfied the inclusion criteria, 11 used Arabica green coffee, three used Robusta, and five included both species. The reported postharvest processing methods include dry^22,40^, semi-dry^40,41^, and wet processing^19,20,26,33,41^. Regarding roasting methods and roast profiles, five studies used drum roasters (non-isothermal) with temperatures ranging from 180 to 220 °C and roast times of 4 to 33 min^17,20,40,42,43^. Nine studies conducted isothermal roasting at constant temperatures between 160 and 300 °C from 10 min to 24 h, using various equipment such as small-scale fluidized bed laboratory roasters^19,26, ^drying ovens^18,24,28^, microwaves^24^, infrared ovens^24^, pans^44^, and air fryers^44^. The color-measuring instruments used in all the above-mentioned studies included various spectrophotometers and colorimeters with varying specifications, such as different aperture sizes, observation angles, and illuminants (Table 4). 

**Fig. 6:**
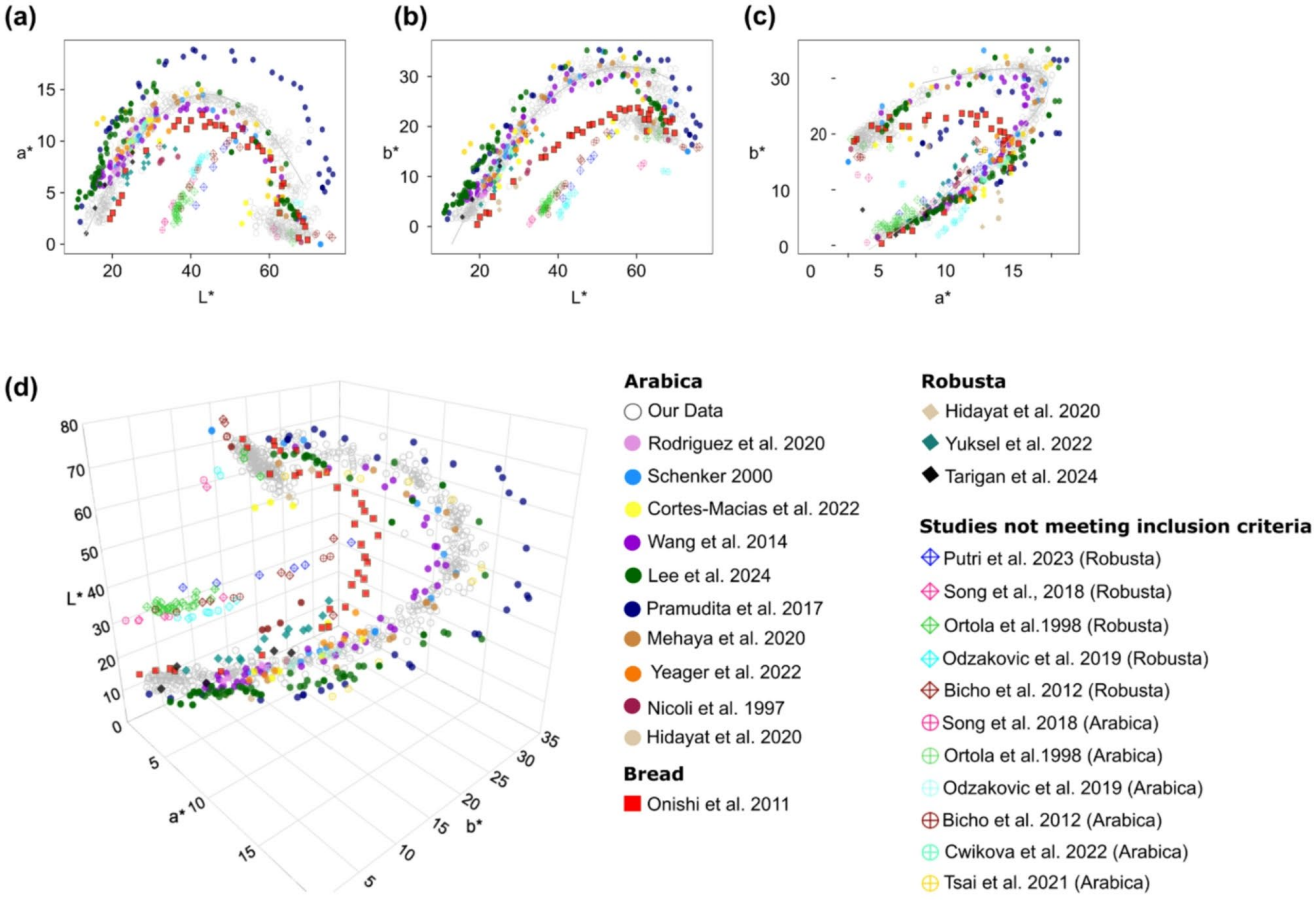
Direct comparison of our L*a*b* values with data from other publications. (**a**) a* vs. L*, (**b**) b* vs. L* (**c**) a* vs. b*, and (**d**) Three-dimensional plot of the L*a*b* values.

In total, these publications include 392 distinct L*a*b* values. Figure 6 shows scatter plots of our L*a*b* values superimposed with data from the 20 publications. The gray open circles represent our measured L*a*b* values (also shown in Fig. 5), while the filled colored markers correspond to L*a*b* values from each publication, with each color indicating a different study. Circles denote Arabica coffee, diamonds represent Robusta, and squares indicate data from the bread study. The colored circle-plus and diamond-plus symbols highlight Arabica and Robusta data, respectively, from the seven studies that did not meet our criteria but were included for the sake of completeness since they suggest a potentially interesting trend.

Focusing first on the 12 studies that met our inclusion criteria, we observed little differences in color dynamics between Arabica and Robusta coffee, as L*a*b* values from publications using Robusta coffee aligned with the coffee color curve (Fig. 6)^17,24,45^. Similarly, the various post-harvest processing methods did not appreciably influence the dynamics of color during roasting^40,42^. Despite the wide range of reported roasting conditions and roast profiles, most L*a*b* values qualitatively accord with the color curve. An exception is noted in the work by Pramudita et al.,^18^ which reported consistently higher a* values, shown by the navy blue points in (Fig. 6). This study, however, employed a very unusual roasting technique: they baked the beans for 24 h in an oven, suggesting that the differences in a* might result from that long baking time.

Interestingly, five of the seven studies that did not meet our inclusion criteria—represented by circle-plus and diamond-plus markers in Fig. 6—did not align with our color curve but instead formed a distinct cluster, while two aligned with the color curve. This distinct cluster of the five independent studies follows a similar slope of a* versus L* (slope = 0.48 in the range of L* = 32.1 to 49.8) compared to our fitted curve (slope = 0.46 in the range L* = 10.97 to 39.86) with L* values approximately 20 points higher than our curve. Surprisingly, L*a*b* values from the bread-baking study^34^ also qualitatively followed the coffee color curve, suggesting that the roasted arabica coffee color curve model may extend to other food processing methods involving Maillard reactions, such as bread-baking.

The average ΔE* values, which ranged from 0.81 to 18.91, are shown in (Table 4). Ten selected studies had average ΔE* values below 4, indicating minimal differences between their reported color values and ours. Four studies had ΔE* values between 5 and 8, indicating noticeable differences, while five studies had an average ΔE* value of 16.25, indicating substantial deviations in color. Notably, the bread-baking study had a ΔE* mean of approximately 8. These findings suggest that the color curve model can predict color across different coffee types and roast profiles during roasting, establishing it as a “universal roasted arabica coffee color curve”.

**Table 4:** Sources of selected publications on the dynamics of coffee color during roasting, including coffee origin, roast profiles, and type of color-measuring instrument used. The final column reports the average ΔE* between equations (2) and (3) and with the L*a*b* values reported in the respective study.

Reference	Coffee type/origin/postharvest processing method	Roasting equipment /batch size	Roast profiles	Instrument/ Illuminant /aperture size/observation angle	Mean ΔΕ*
Tarigan et al.^20^	Robusta/Indonesia/ Wet processing	Probat BRZ 2/100 g	Temp:180, 190, 220, 200 ℃ Time: 4, 6, 7, 8 min	Hunterlab mini scan EZ/unspecified/unspecified/[45/0°]	0.81
Schenker^26^	Arabica/Colombia /Wet processing	Fluidized-bed laboratory roaster/100 g	HTST: 260℃, 180s LTLT: 220℃, 720s	Konica minolta chroma meter CR-310/unspecified/50 mm/[d/0°]	1.07
Rodriguez et al.^41^	Arabica/Colombia/ Wet, Semi-dry processing	TC 150R laboratory quantik roaster /150 g	Temp:180–220 °C Time:7–33 min	Konica minolta chroma meter CR-410 /unspecified/50 mm/[d/0°]	1.38
Yeager et al.^33^	Arabica/ El Salvador, Indonesia, Ethiopia/ wet, wet-hulled & honey processing	Probatino P5/ unspecified	Temp:185–220 °C Time:11–15 min	Konica minolta chroma meter CR-400/D65/50 mm/[d/0°]	1.51
Cwikova et al.^22^	Arabica/14 origins/ wet, dry processing	Unspecified/ unspecified	Temp:194–197 °C (light), 202–209 °C (medium), 212–217 °C (dark)	Konica minolta chroma meter CR-3500d/unspecified/unspecified	1.69
Wang et al.^19^	Arabica/Brazil/wet processing	Fluidized bed roaster-fresh roast SR 500/45 g	Temp:210, 220, 230,240 °C Time: start & end of 1st & 2nd crack, 48 s after 1st & 2nd crack	Konica minolta chroma meter CR -3500d /unspecified/30 mm/[d/8°]	1.70
Mehaya et al.^28^	Arabica/Ethiopia/ unspecified	Drying oven-heratherm OMS60, /100 g	Temp:160, 180, 220 °C Time:10, 20, 30, 40 min	Hunter, lab scan XE/unspecified/unspecified/[45/0°]	1.77
Cortes-Macias et al.^40^	Arabica/Colombia/ dry, wet, semi-dry processing	TC Rotary drum roaster/150 g	Temp:190 ± 2.5 °C Time:8.24, 9.12 min	Konica minolta chroma meter CR-700d/D65/unspecified/[d/8°]	2.87
Yuksel et al.^24^	Robusta/ unspecified/ unspecified	Microwave, Infrared oven, drying oven/100 g	Temp: 700, 490, 350 W (microwave); 600, 1200 W(infrared); 160, 180, 200,220 °C (oven) Time: 10, 20, 30 min	HunterLab colorflex EZ/D65/unspecified/unspecified	3.0
Lee et al.^44^	Arabica/Brazil/ unspecified	CBR-101 A hot air roaster, pan, air fryer/ 100 g	Temp: 200, 220, 240 °C, Time: 1, 3, 6, 12, 15, 18, 21, 24, 27, 30 min	NR-12 A color meter/unspecified/unspecified/ unspecified	3.71
Tsai et al.^8^	Arabica/Indonesia/unspecified	Unspecified/ unspecified	Temp:200 °C	HunterLab mini scan EZ, 4000 S/unspecified/unspecified/[45/0°]	5.02
Pramudita et al.^18^	Arabica /Colombia/unspecified	Drying oven/5.5 g	Temp: 140, 180, 220, 260, 300 °C Time:15,30,60,120,240 min, 24 h	NF-333 handy spectrophotometer/unspecified/8 mm/[45/0°]	5.03
Hidayat et al.^17^	Arabica, Robusta/unspecified/wet processing	Drum roaster/750 g	Temp: 180 °C Time: few mins post 1st & 2nd crack	NH 310 colorimeter/unspecified/unspecified /[d/8°]	6.79
Nicoli et al.^56^	Arabica/unspecified/ unspecified	VTRV laboratory roaster/1000 g	Temp: 200,220 °C Time: 8,10,15,20 min	Konica minolta chroma meter CR-200/unspecified/unspecified/unspecified	7.34
Bicho et al.^1^	Arabica, Robusta/Brazil, India/ unspecified	Unspecified/ unspecified	Temp: 200–240 °C Time: 5–12 min	Konica minolta chroma meter CR-300/D65, C/8 mm /[d/0°]	14.51
Song et al.^43^	Arabica, Robusta/ Guatemala, India/ unspecified	OKS-1.5 drum roaster/ unspecified	Temp: max 220 °C Time: 11–13 min	HunterLab ultra scan XE/unspecified/unspecified/unspecified	15.28
Ortolá et al.^23^	Arabica, Robusta/ Colombia, Indonesia/ unspecified	Tec 250 especial C drum roaster /25 g	Temp: 200,235,250,265,280,295 °C Time:5–30 min	HunterLab ultra scan XED65/unspecified/[d/0°]	15.93
Putri et al.^21^	Robusta/ unspecified/ unspecified	Unspecified/ unspecified	Unspecified	3NH-NH300 colorimeter/unspecified/unspecified/unspecified	16.63
Odzakovic et al.^16^	Arabica, Robusta/India/ unspecified	Unspecified/ unspecified	Temp:167, 171, 175 °C Time: 25 min	Konica minolta chroma meter CR-410 /D65 /50 mm/[d/0°]	18.91
Onishi et al.^34^	Bread/pullman-type bread	DOE-02 static electric oven	Temp:140–260 ℃ Time: 5–80 min	NF-333 pen-type spectrophotometer	8.0

## Discussion

This study aimed to investigate how roast profiles and coffee origins influence the color of coffee during commercial-scale roasting. Our results show that roast profiles significantly affect color dynamics during roasting, with profiles with higher RORs resulting in faster rates of color change (Figs. 2 and 3a). This result confirms that the application of specific roast profiles allows roasters to effectively control color development during roasting, allowing them to achieve the desired roast levels. Generally, our results support established trends in roast color development while encompassing a diverse range of industry-standard roast profiles and commercial-scale roasting applications^26,46^.

During roasting, the L*a*b* values first increased until the color change to the first crack phase, then decreased towards the end of the roast (Fig. 3a). The observed changes in color can be attributed to melanoidin formation resulting from nonenzymatic browning reactions such as the Maillard reaction^47,48^. A key finding in this study was that regardless of roast profile or coffee origin, the coffees always had approximately the same L*a*b* values at the major roast milestones, including color change, the first, and second crack (Fig. 4). This finding indicates that the color observed at these significant roast milestones can be used as key parameters for evaluating and standardizing the degree of roast. The L* values observed at these roast milestones (29.74 ± 1.9 at the first crack and 19.96 ± 1 at the second crack) are consistent with those reported by Wang and Lim (2012), who reported average L* values of 25–28 at the first crack and 20 at the second crack^27^. As shown in Fig. 3b, roast color (L* and a* coordinates) varied significantly among the three coffee origins for specific roast profiles (SS and EM). A potential reason is that the composition of melanoidins, which are responsible for the color formation, depends on polysaccharides, amino acids, proteins, and phenolic compounds (chlorogenic, caffeic, or ferulic acids) present in coffee^49–51^and these components can vary based on the green coffee origin. These results are comparable to those of Rodriguez et al. (2020), who observed significant color differences in roasted coffee between wet and semi-dry processing methods^41^.

Perhaps the most surprising result presented here is that, regardless of the wildly different roast profiles and wildly different green coffee beans, all our experimental measurements plotted in the L*a*b* color space followed what we refer to as “the universal roasted arabica coffee color curve” (Fig. 5). Polynomial regression results showed strong correlations among the L*a*b color coordinates, indicating that one coordinate can effectively be used to predict another. Similar quadratic models for correlations among L*a*b* coordinates have been reported by Onishi et al. (2011) for white bread baking and Pramudita et al. (2017) for isothermal coffee roasting in a drying oven^18,34^. Our study, however, extends these findings to diverse non-isothermal roast profiles and commercial-scale roasting, using coffee from various origins and postharvest processing methods.

As observed in Fig. 6, when we compared the coffee color curve to experimental data from existing publications, most roast color data closely followed the curve. The high ΔE* values (> 15) of the five studies that did not follow the color curve can potentially be attributed to several factors, including differences in measuring instruments and settings, roasting methods, sample preparation, data processing, and reporting. We emphasize that even for measurements ostensibly using the same L*a*b* color space, the data reported in different publications using different measuring devices can vary considerably. Factors such as illumination, viewing geometry, and aperture size can influence color measurements, complicating comparisons between studies^10,52^. As shown in Table 4, three of the five studies^1,16,21^ did not report their roasting methods or roast profiles, while two did not specify their colorimetry settings^1,21^. Qualitatively, the reported color values of these five studies appear, compared to our experience with roasted coffee, unusually gray or whitish, a characteristic that to our knowledge is not typically observed in roasted coffee. Notably, one study^16^ reported a maximum roasting temperature of 175 °C, which is below the typical first crack temperature (~ 196 °C), suggesting that their coffee may have been significantly underdeveloped. Interestingly, all five studies that deviated from our color curve included Robusta coffee. However, since other Robusta data points from different studies aligned with our curve, the coffee species alone is unlikely to account for these discrepancies.

Overall, the universal roasted coffee color curve offers significant implications for the coffee industry by providing a precise, quantitative standard for defining roast levels. Currently, there are no universally accepted industry standards for what is meant by “light roast,” “medium roast,” or “dark roast,” despite the importance of these terms in marketing and consumer acceptance^53^. Some roasters produce ‘light roasts’ that are darker than the ‘dark roasts’ produced by others, leading to consumer confusion. Various roast analyzers use different scales to report roast levels, including Agtron™, Color Track, Difluid, Roastvision, Roastpic, and Colorette. As a result, a coffee rated 40 on Agtron’s commercial scale may not correspond to the same value on other devices^52^. The existence of a universal roasted arabica coffee color curve and the uniformity of color at key roast milestones greatly simplify efforts to develop a standardized nomenclature based on quantitative measurements of color. For example, the curve can be divided into ranges of L*a*b* values corresponding to commonly used roast levels, such as light, medium, and dark. Once defined, these ranges could serve as thresholds for classifying any roasted coffee sample based on its location on the curve. However, assigning specific cutoff values requires input from coffee industry professionals and consumers, since any such dividing line is ultimately arbitrary and is best determined by industry consensus and/or surveying a statistically meaningful number of consumers. Preliminary efforts toward this goal are described in Ristenpart et al.^53^. Furthermore, by dividing the universal roasted coffee color curve into sections based on the major roast milestones, the curve could also guide the use of plain language descriptions for the colors observed along the curve (e.g., medium brown, reddish brown, etc.), which would also facilitate communication of roast level to other coffee industry members and consumers.

Although we refer to the roasted arabica coffee color curve as “universal” here, a few caveats are in order. We focused on high-quality, specialty-grade arabica coffees that were relatively free of defects; it is possible that lower-grade coffees with high fractions of coffee defects, such as blacks and sours, may show different color dynamics on average. We also did not investigate decaffeinated coffee, which is known to be lighter in color when green and responds to roast profiles differently^54^. Green coffee is also known to change color as it ages and although our differently colored green coffees all fell onto the universal curve during roasting, that observation does not preclude the possibility that other types of green coffee (e.g., very fresh or very old) might show/exhibit different color dynamics. We also did not perform any experimental work on robusta or *C*. *liberica* coffee, the other two predominant species of commercially cultivated coffee. However, our meta-analysis indicated that robusta coffee follows the universal roasted coffee color curve^55^.

## Conclusion

To the best of our knowledge, this study is the first to systematically investigate the dynamics of coffee color during roasting using industry-standard roast profiles and various coffee origins on a commercial scale. Overall, our results demonstrate that changes in coffee color during roasting follow a consistent path in the CIELAB color space, which we define as “the universal roasted arabica coffee color curve.” Furthermore, regardless of the roast profile or origin, our coffees consistently exhibited approximately the same L*a*b* values at significant roast milestones such as color change, first crack, and second crack. The universal roasted arabica coffee color curve provides a valuable quantitative standard for defining roast levels in the coffee industry. Future work should explore a broader range of coffee species, origins, and postharvest processing methods—including decaffeinated coffee—as well as other roast profiles, including shorter roast times. Additionally, future studies should establish correlations between the CIELAB color space and roast level measurement scales, including Agtron, Colorette, and Colortrack, to ensure the curve’s applicability across different color-measuring instruments. Further research should also explore the correlation between chemical composition and color development during coffee roasting.

## Electronic supplementary material

Below is the link to the electronic supplementary material.

Supplementary Material 1

## Data Availability

All relevant data are available from the corresponding author upon request.
