# Correction: A universal color curve for roasted arabica coffee

**DOI:** 10.1038/s41598-025-23127-3

**Published:** 2025-10-10

**Authors:** Laudia Anokye-Bempah, Timothy Styczynski, William D. Ristenpart, Irwin R. Donis-González

**Affiliations:** 1 Department of Biological and Agricultural Engineering, University of California Davis, 3024 Bainer Hall, Davis, CA 95616 USA; 2 Coffee Center, University of California Davis, Davis, CA 95616 USA; 3 Bridge Coffee Co., Marysville, CA 95901 USA; 4 Department of Chemical Engineering, University of California Davis, Davis, CA 95616 USA

Correction to: *Scientific Reports* 10.1038/s41598-025-06601-w, published online 07 July 2025

The original version of this Article contained errors.

In Figure 4, the X-axis labels for panels A, B and C were incorrectly given as ‘Roast phase’.

The original Figure 4 and accompanying legend appear below.

**Fig. 4:**
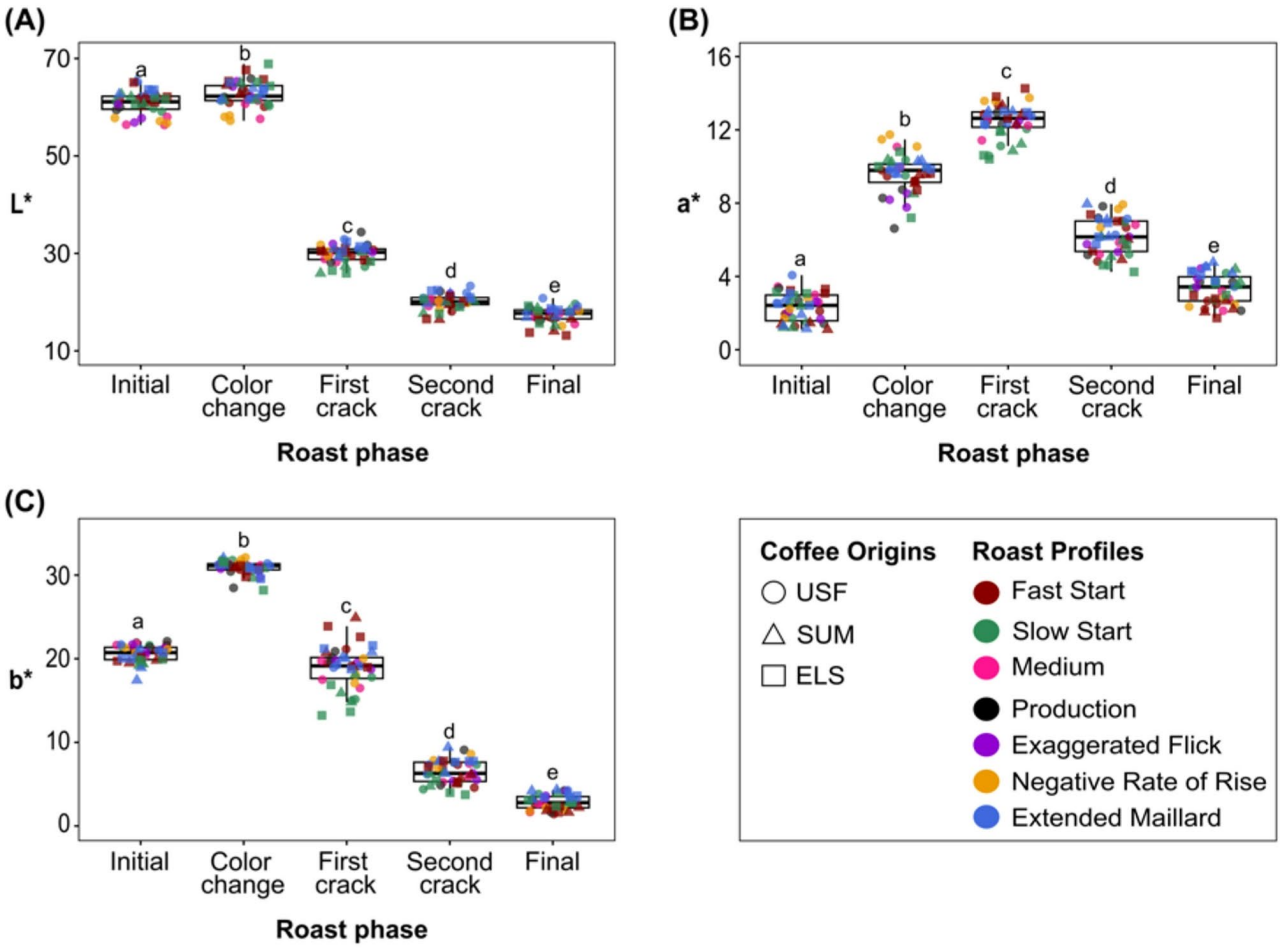
Boxplots showing the distributions of L*, a*, and b* values (**A**–**C**, respectively) at each roast milestone for all seven roast profiles and three coffee origins. The bottom and top edge of the boxes represent the 25 and 75th percentiles, respectively, the line inside the box represents the median, and the whiskers denote the range of the observed values. Lowercase letters indicate statistically significant differences among roast milestones according to Tukey HSD test.

In addition, in the Discussion section, the citation of References 53–55 was incorrect.

As a result,

“Preliminary efforts toward this goal are described in Ristenpart et al.^54^. Furthermore, by dividing the universal roasted coffee color curve into sections based on the major roast milestones, the curve could also guide the use of plain language descriptions for the colors observed along the curve (e.g., medium brown, reddish brown, etc.), which would also facilitate communication of roast level to other coffee industry members and consumers.

Although we refer to the roasted arabica coffee color curve as “universal” here, a few caveats are in order. We focused on high-quality, specialty-grade arabica coffees that were relatively free of defects; it is possible that lower-grade coffees with high fractions of coffee defects, such as blacks and sours, may show different color dynamics on average. We also did not investigate decaffeinated coffee, which is known to be lighter in color when green and responds to roast profiles differently^55^. Green coffee is also known to change color as it ages and although our differently colored green coffees all fell onto the universal curve during roasting, that observation does not preclude the possibility that other types of green coffee (e.g., very fresh or very old) might show/exhibit different color dynamics. We also did not perform any experimental work on robusta or *C*. *liberica* coffee, the other two predominant species of commercially cultivated coffee. However, our meta-analysis indicated that robusta coffee follows the universal roasted coffee color curve.”

now reads:

“Preliminary efforts toward this goal are described in Ristenpart et al.^53^. Furthermore, by dividing the universal roasted coffee color curve into sections based on the major roast milestones, the curve could also guide the use of plain language descriptions for the colors observed along the curve (e.g., medium brown, reddish brown, etc.), which would also facilitate communication of roast level to other coffee industry members and consumers.

Although we refer to the roasted arabica coffee color curve as “universal” here, a few caveats are in order. We focused on high-quality, specialty-grade arabica coffees that were relatively free of defects; it is possible that lower-grade coffees with high fractions of coffee defects, such as blacks and sours, may show different color dynamics on average. We also did not investigate decaffeinated coffee, which is known to be lighter in color when green and responds to roast profiles differently^54^. Green coffee is also known to change color as it ages and although our differently colored green coffees all fell onto the universal curve during roasting, that observation does not preclude the possibility that other types of green coffee (e.g., very fresh or very old) might show/exhibit different color dynamics. We also did not perform any experimental work on robusta or *C. liberica* coffee, the other two predominant species of commercially cultivated coffee. However, our meta-analysis indicated that robusta coffee follows the universal roasted coffee color curve^55^.”

The original Article has been corrected.

