# Supplementary material for: A universal color curve for roasted arabica coffee
Source: Sci Rep. 2025 Jul 7;15:24192. doi: 10.1038/s41598-025-06601-w (PMC12234775; doi:10.1038/s41598-025-06601-w)
Supplement: Supplementary file 1 — Supplementary Material 1 [file 41598_2025_6601_MOESM1_ESM.pdf]

Supplementary Information:

A Universal Color Curve for Roasted Coffee

Laudia Anokye-Bempah, Timothy Styczynski, William D. Ristenpart,

& Irwin R. Donis-González

Figure S1. Computer Vision System

Figure S2. PRISMA flow diagram for literature search and data extraction

Figure S3. Histogram of ΔE* values between individual datapoints and the universal color curve

Figure S4: Residual Analysis for Mixed-Effects Regression

Table S1. Table of F-ratios from the mixed ANOVAs for coffee origins

Figure S1. Computer Vision System for Photographing Samples

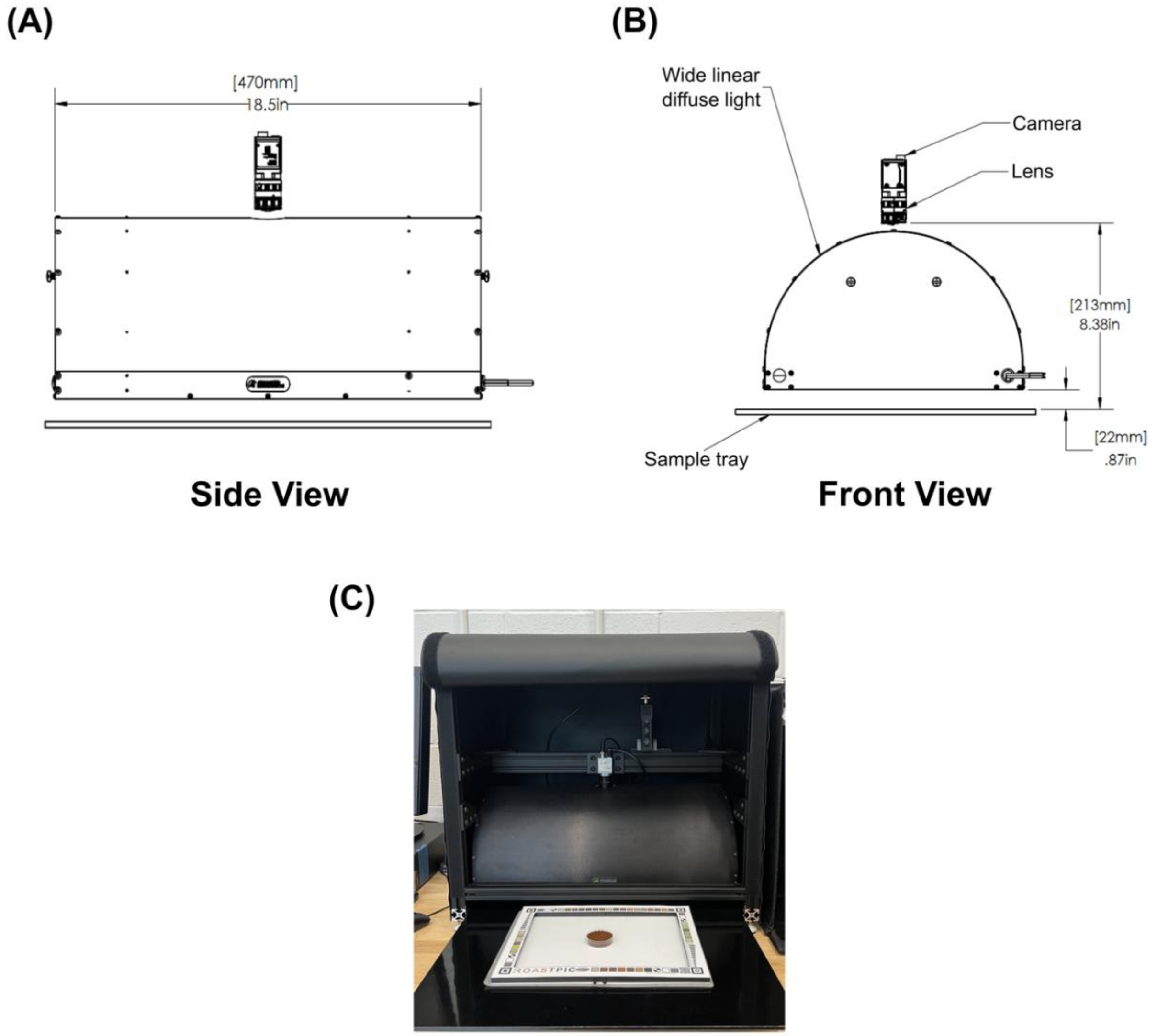

Supplementary Figure S1. Color Vision System for photographing coffee samples. A) side view, B) front view, and C) photo showing a ground coffee sample placed in a petri dish on the sample tray before measurement.

Figure S2. PRISMA flow diagram for literature search and data extraction.

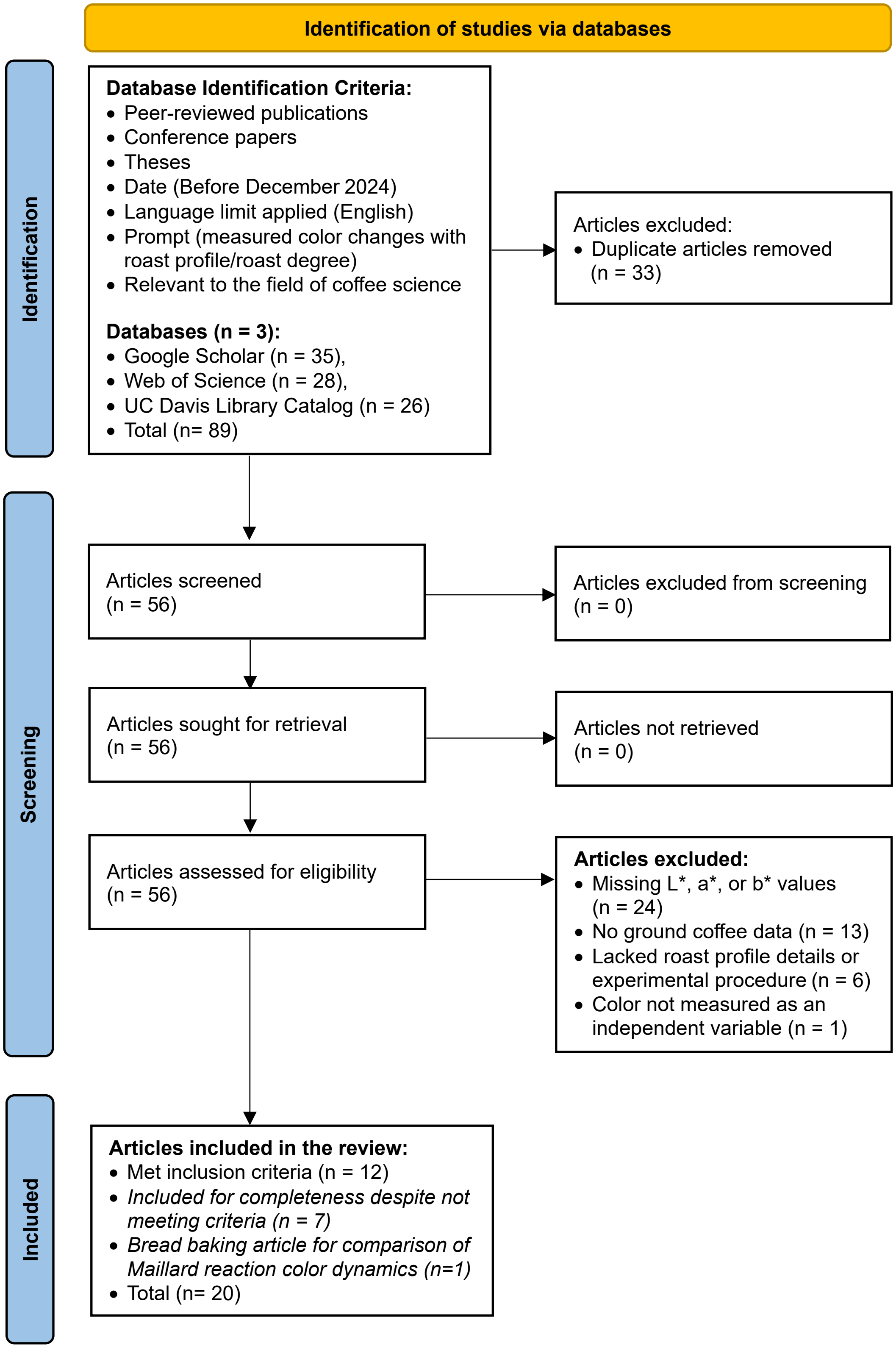

Supplementary Figure S2. PRISMA (Preferred Reporting Items for Systematic Reviews and Meta-Analyses) flow chart sequencing the review process

Figure S3: Histogram of ΔE* values between our individual experimental measurements of L*, a*, and b* coordinates and the universal color curve (cf. equations 2 and 3 in the main text).

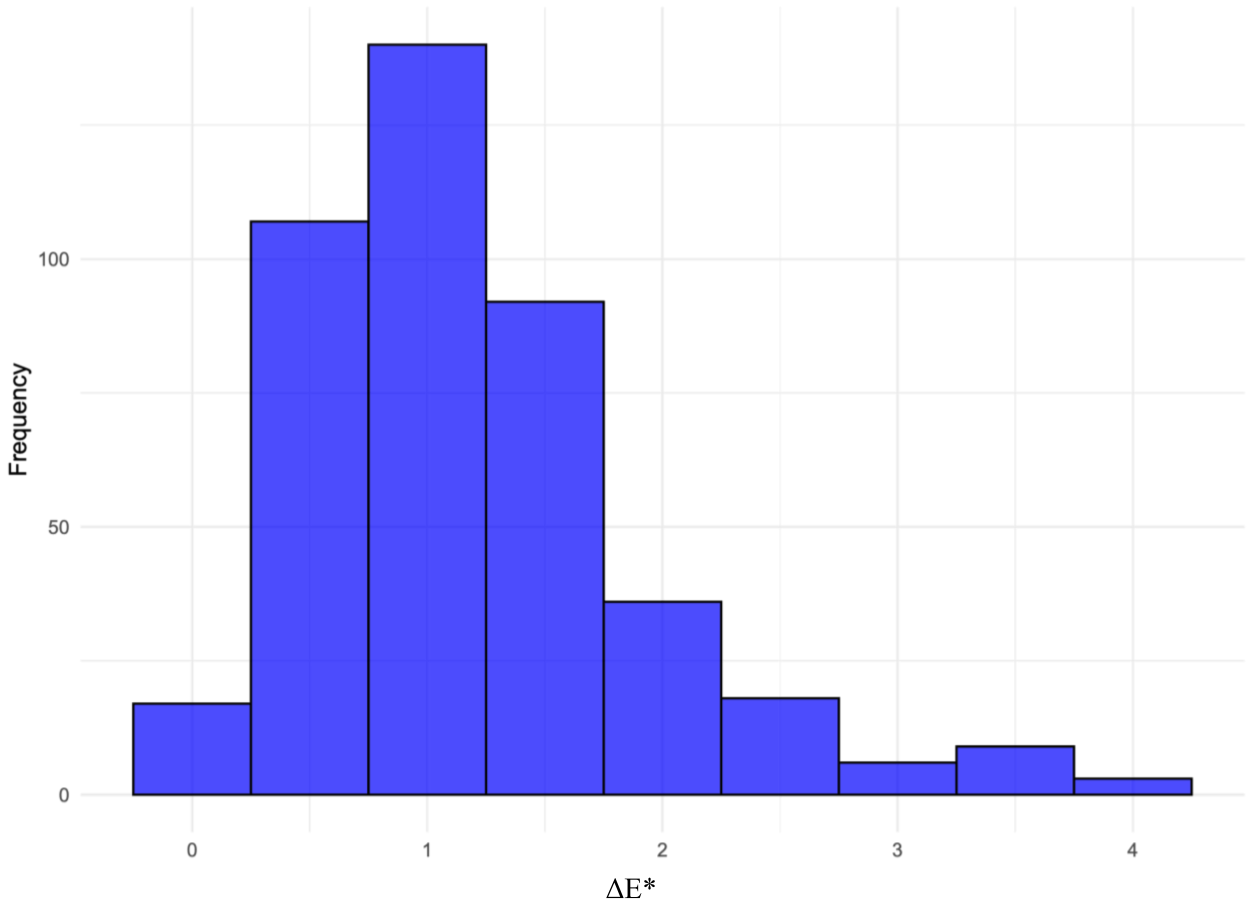

Figure S4: A) Residual Analysis for mixed-effects regression (b vs L)

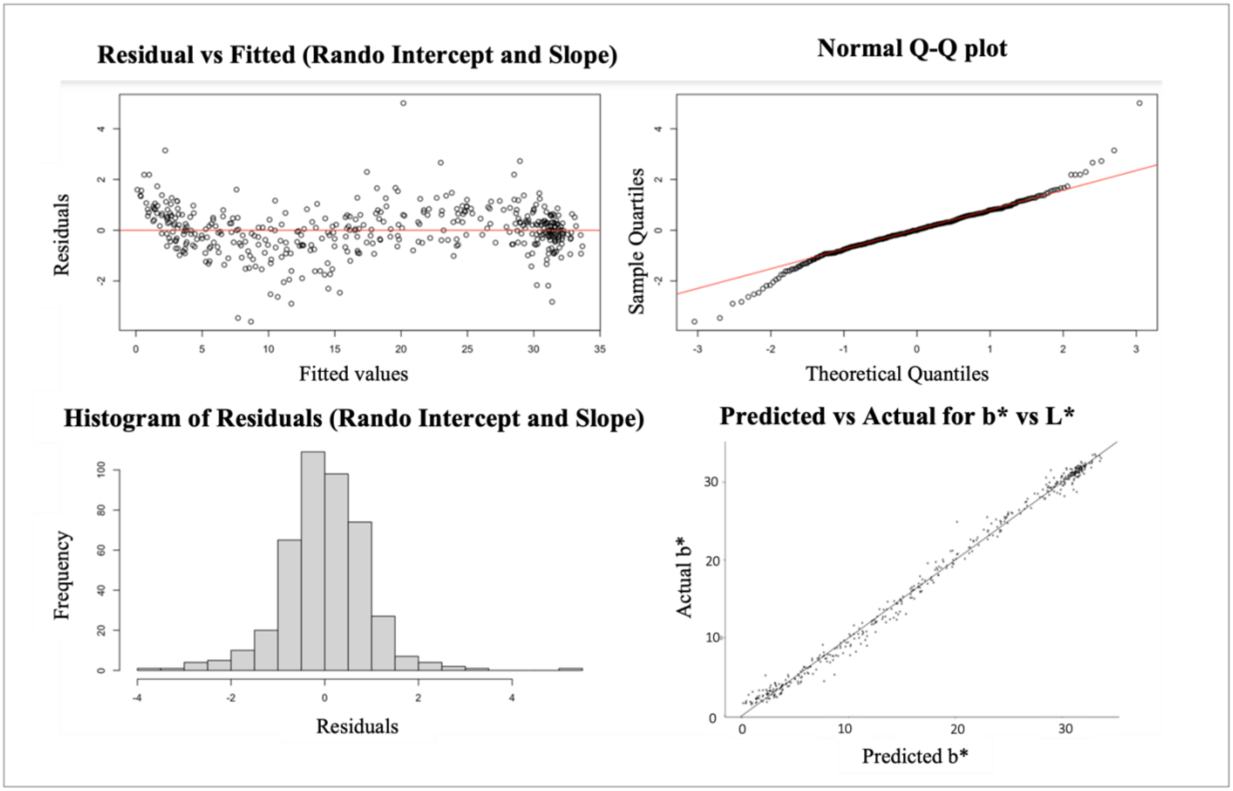

B) Residual Analysis for mixed-effects regression (a vs L)

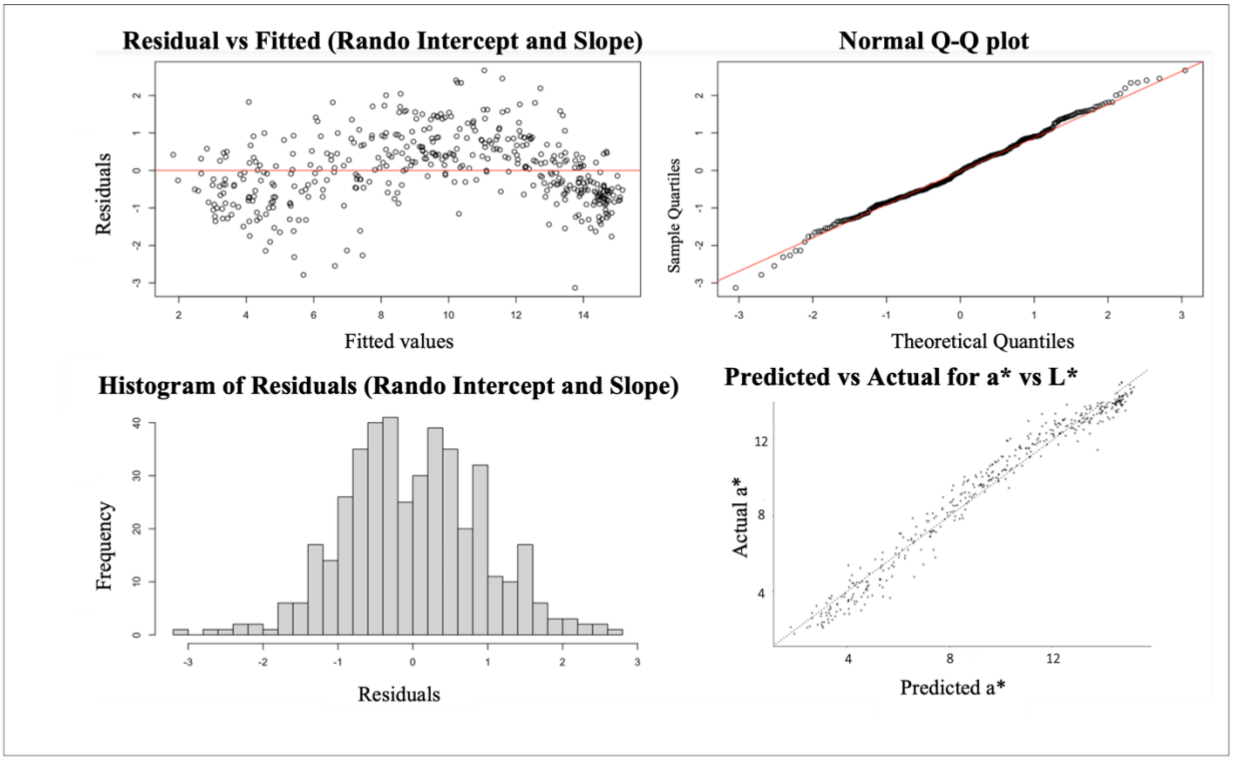

Table S1: Table of F-ratios from the mixed ANOVAs for coffee origins, significance indicated by * (α = 0.05), with corresponding degrees of freedom (df) and p-values (df values were corrected using Greenhouse-Geisser estimates due to a violation of sphericity indicated by Mauchly's test).

Fast Start Profile

Parameter	Factor	df	F-ratio	P-value
L*	Time	2.56	2633.69	<0.001*
	Time* Origin	5.12	5.49	<0.001*
	Origin	2	3.51	0.098
a*	Time	2.036	992.88	<0.001*
	Time* Origin	4.072	1.982	0.160
	Origin	2	3.157	0.513
b*	Time	2.492	1356.02	<0.001*
	Time* Origin	4.984	3.343	0.032
	Origin	2	.751	0.200

Slow Start Profile

Parameter	Factor	df	F-ratio	P-value
L*	Time	3.226	1680.751	<0.001*
	Time* Origin	6.452	2.322	0.071
	Origin	2	3.512	0.098
a*	Time	2.486	667.540	<0.001*
	Time* Origin	4.972	1.351	0.297
	Origin	2	3.850	0.562
b*	Time	2.492	1356.018	<0.001*
	Time* Origin	4.984	3.343	0.032
	Origin	2	.751	0.200

Extended Maillard Profile

Parameter	Factor	df	F-ratio	P-value
L*	Time	3.795	2831.512	<0.001*
	Time* Origin	7.590	5.009	0.001*
	Origin	2	11.484	.009
a*	Time	3.252	1435.479	<0.001*
	Time* Origin	6.504	2.448	0.059
	Origin	2	1.001	.422
b*	Time	3.346	1387.405	<0.001*
	Time* Origin	6.691	3.491	0.014
	Origin	2	.156	.859
